# Changes in real-world walking speed following 60-day bed-rest

**DOI:** 10.1038/s41526-023-00342-8

**Published:** 2024-01-13

**Authors:** Marcello Grassi, Fiona Von Der Straten, Charlotte Pearce, Jessica Lee, Marcin Mider, Uwe Mittag, Wolfram Sies, Edwin Mulder, Martin Daumer, Jörn Rittweger

**Affiliations:** 1 Sylvia Lawry Center for Multiple Sclerosis Research e.V., Munich, Germany; 2 Institute of Aerospace Medicine, Department of Muscle and Bone Metabolism, German Aerospace Center, Cologne, Germany; 3 TUM School for Computation, Information and Technology, Technical University of Munich, Munich, Germany; 4 Trium Analysis Online GmbH, Munich, Germany; 5 Department of Pediatrics and Adolescent Medicine, University Hospital Cologne, Cologne, Germany

**Keywords:** Predictive markers, Physiology

## Abstract

The aim of this work was to explore whether real-world walking speed (RWS) would change as a consequence of 60-day bed-rest. The main hypothesis was that daily RWS would decrease after the bed-rest, with a subsequent recovery during the first days of re-ambulation. Moreover, an exploratory analysis was done in order to understand whether there is an agreement between the loss in RWS after bed-rest and the loss in the maximum oxygen uptake capacity (VO_2max_), or the loss in maximal vertical jump power (JUMP) respectively. Twenty-four subjects were randomly assigned to one of three groups: a continuous artificial gravity group, an intermittent artificial gravity group, or a control group. The fitted linear mixed effects model showed a significant decrease (*p* < 0.001) of RWS after the 60-day bed-rest and a subsequent increase (*p* < 0.001) of RWS during the 14-day recovery period in the study facility. No or little agreement was found between the loss in RWS and the loss in VO_2max_ capacity or the loss in maximal vertical jumping power (RWS vs. VO_2max_: *p* = 0.81, RWS vs. JUMP: *p* = 0.173). Decreased RWS after bed-rest, with a follow-up recovery was observed for all three groups, regardless of the training intervention. This suggests that RWS, also in these settings, was able to reflect a de-conditioning and follow-up recovery process.

## Introduction

The physiological responses of humans exposed to microgravity have been of particular interest since the beginning of human spaceflight. The conditions in space cause severe adaptations in sensorimotor, cardiovascular and neuromuscular systems of astronauts^1^. To safeguard astronauts’ health, it is essential to identify functionally relevant declines in fitness levels upon their return to Earth in order to facilitate a fast and full recovery.

Generally, gait speed has been recognized as an informative functional test to assess a person’s health^2–5^. Slow gait speed, for instance, is an indicator of decreased functionality, and even of mortality in older adults^6,7^. Many factors are likely involved in decreases in gait speed. To name a few; muscular factors including loss of motor units, decrease in muscular contraction speed and velocity, disrupted muscular activation, and neurological factors including decrease in nerve conduction velocity, decrease in the reaction time and other diseases related with the central and peripheral nervous systems^8–11^. A systematic review stated that “gait speed at usual pace was a strong and consistent predictor of adverse health outcomes, and gait speed as a single-item tool was at least as sensible as the composite tools in predicting these outcomes over time” in older adults^12^.

Bed-rest is a well-accepted model to simulate the impact of spaceflight on human physiology,^1^. Immobilization by bed-rest leads to a rapid de-conditioning process in most bodily organs^13^. Eight weeks of strict bed-rest lead to a reduction in calf muscle size by ~20%^14^, to a similar reduction in plantar flexor muscle strength and a decline in peak jumping power of ~30%^15^. It can only be assumed that such musculoskeletal de-conditioning would affect a person’s ability to locomote rapidly. Previous work involving gait course analyses after 60 days of bed-rest showed that preferred walking speed is impaired up to 7 days after re-ambulation^16^.

In previous bed-rest studies, individual fitness levels were mainly assessed by using maximal effort tests^17^. However, measurements were only taken at discrete points in time and only until 2 weeks after the end of bed-rest. It is known that discrete measurements as gait tests have a high day-to-day variability^18,19^, thus a continuous and long-term measurement of RWS can provide additional insight in the recovery process after bed-rest. Moreover, it is well known now that gait speed of subjects who are observed under artificial conditions significantly differs from their self-selected gait speed^20–24^. In a previous study^21^, where walking behavior was investigated during the recovery after total hip replacement surgery, patients exaggerated their walking speed while under observation compared to their natural behavior at home. This seems to suggest that longer monitoring periods in an unobserved environment would add valuable information on functional fitness.

The main aim of this study was to investigate if and how RWS is affected by 60 days of bed-rest, and whether RWS can be used as an indicator of fitness status. The main hypothesis was that a decrease in walking speed would occur after the bed-rest period, with a subsequent fast recovery towards RWS values observed before the bed-rest period. Moreover, effects of training interventions on RWS across the different study groups were also analyzed.

Lastly, an exploratory analysis was done to explore whether changes in RWS are associated to other more commonly used maximal physical assessments (VO_2max_ measurements and maximal vertical jump tests), to understand whether there is agreement in the outcomes between RWS and the other tests, or if there is a generally poor agreement, suggesting that they measure something complementary to each other.

## Results

### Data completeness

Out of the 120 expected periods (5 periods × 24 subjects), 112 periods were captured. For two subjects, measurements were not collected for either the *pre.home* or the *pre.dlr* periods, making it impossible to establish a baseline measurement for RWS. Thus, it was decided to remove those two subjects from the dataset since no before/after bed-rest comparison was possible. For three other subjects, measurements from the *pre.home* period were not collected, and for one subject measurements for the *post.home(R* + *90)* period were not collected. Those four subjects were not excluded from the dataset since the *pre.dlr* period was available, and thus the before/after comparison was still possible.

### Randomness of incomplete observations

Out of the 944 recording days, 182 (17%) had a wearing time smaller than 10 h per day (mean ± sd: 5.76 ± 2.52). From visual inspection of the Q-Q plot (see Supplementary Methods for further details on the method employed to establish validity of measurements with <10 h of recording per day) it was seen that most of the cumulative distribution function of the average walking speed are closely distributed according to a uniform distribution on [0,1], suggesting that the data were missing at random.

### Wearing time

Wearing time, or adherence, was generally very high throughout all the study phases (h/day; mean ± sd): *pre.home*: 14.2 ± 4.1; *pre.dlr*: 13.5 ± 3.5; *post.dlr*: 12.9 ± 3.5; *post.home(R* + *28)*: 11.3 ± 5.1; *post.home(R* + *90)*: 12.7 ± 5.1. Data about wearing time by group and period are summarized in Table 1.

**Table 1:** mean ± sd of daily RWS, walking distance and wearing time per period and per group.

	*pre.home*	*pre.dlr*	*post.dlr*	*post.home(R* + *28)*	*post.home(R* + *90)*
group	Walking speed (m/s)				
**Ctrl**	0.93 ± 019 (−0.01)	0.94 ± 0.07 (–)	0.82 ± 0.09 (−0.12)	0.95 ± 0.22 (0.01)	0.95 ± 0.16 (0.02)
**iAG**	0.94 ± 0.18 (−0.03)	0.96 ± 0.07 (–)	0.86 ± 0.08 (−0.11)	0.98 ± 0.18 (0.01)	0.96 ± 0.17 (−0.01)
**cAG**	1.00 ± 0.3 (0.02)	0.98 ± 0.12 (–)	0.83 ± 0.09 (−0.15)	1.04 ± 0.23 (0.06)	1.08 ± 0.21 (0.11)
	Walking distance (m)				
**Ctrl**	4862.9 ± 2438.6 (2711.5)	2151.4 ± 1148.3 (–)	2525.6 ± 1928.6 (374.2)	3219.8 ± 3309.2 (1068.4)	3841.9 ± 3050.1 (1690.5)
**iAG**	4375 ± 3735.4 (2323.4)	2051.6 ± 975.2 (–)	2306.2 ± 1135.2 (254.6)	4317.1 ± 5637.6 (2265.5)	3707.8 ± 2450.4 (1656.2)
**cAG**	4312.6 ± 2774.2 (2281.4)	2031.2 ± 1010.1 (–)	1997.6 ± 964.5 (−33.6)	3973.2 ± 3623 (1942)	5333.4 ± 4294.5 (3302.2)
	Wearing time (hours/day)				
**Ctrl**	14.1 ± 3.2 (−0.1)	14.2 ± 3.2 (–)	13.2 ± 3.4 (−1)	11.5 ± 4.5 (−2.7)	13.2 ± 6.3 (−1)
**iAG**	14.6 ± 3 (1.6)	13 ± 3.1 (–)	13 ± 2.9 (0)	11.2 ± 5.8 (−1.8)	12.4 ± 4.5 (−0.6)
**cAG**	14 ± 5.5 (0.6)	13.4 ± 4.1 (–)	12.6 ± 4 (−0.8)	11.3 ± 5.1 (−2.1)	12.5 ± 4.4 (−0.9)

In brackets are reported the effect sizes of the differences compared to the *pre.dlr* phase (reference). Bold font is used to represent groups name. Italic font is used to represent names of the study phases.

Group name *Ctrl* refers to the control group, which did not receive any type of intervention to counteract the effects of bed-rest. The group name *iAG* refers to the group that received intermitted artificial gravity training sessions as intervention to counteract the effects of bed-rest. The group name *cAG* refers to the group that received continuous artificial gravity training sessions as intervention to counteract the effects of bed-rest. The phase names refer to the different phases of the study: *pre.home* refers to a 1-week measurement from the 28th to the 21st day at home prior the bed-rest study; *pre.dlr* refers to a 2-week measurement in the DLR ward in the 14 days prior the bed-rest study; *post.dlr* refers to a 2-week measurement in the 13 days after the bed-rest study; *post.home(R* + *28)* refers to a 1-week measurement from the 21st to the 28th day after the bed-rest; *post.home(R* + *90)* refers to a 1-week measurement from the 83rd to the 90th day after the bed-rest.

Summary statistics of real-world walking speed, wearing time and walking distance.

### Linear mixed effect model

**Fig. 1:**
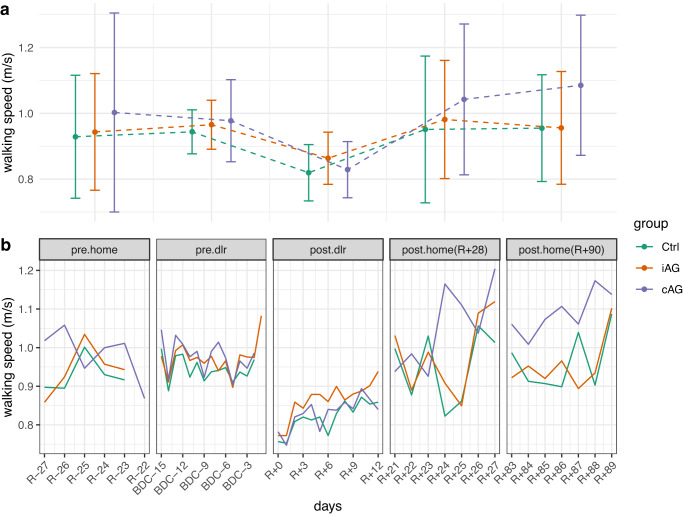
Average real-world walking speed. **a** average RWS values per period per group. Error bars represent ±1 standard deviation. Speed values are expressed in m/s. **b** average RWS values per day per group. Speed values are expressed in m/s. Group name *Ctrl* refers to the control group, which did not receive any type of intervention to counteract the effects of bed-rest. The group name *iAG* refers to the group that received intermitted artificial gravity training sessions as intervention to counteract the effects of bed-rest. The group name *cAG* refers to the group that received continuous artificial gravity training sessions as intervention to counteract the effects of bed-rest. The phase names refer to the different phases of the study: *pre.home* refers to a 1-week measurement from the 28th to the 21st day at home prior the bed-rest study; *pre.dlr* refers to a 2-week measurement in the DLR ward in the 14 days prior the bed-rest study; *post.dlr* refers to a 2-week measurement in the 13 days after the bed-rest study; *post.home(R* + *28)* refers to a 1-week measurement from the 21st to the 28th day after the bed-rest; *post.home(R* + *90)* refers to a 1-week measurement from the 83rd to the 90th day after the bed-rest.

After it was concluded that it was acceptable to include all measurements with wearing time > 1 h per day without introducing a systematic bias in the data, the final dataset resulted in 944 days of measurement with a mean wearing time (mean ± sd) of 13 ± 4.17 h per day (please see Table 1 for details about wearing time per group and study phase). In the lme model significant effects were found in RWS in *post.dlr* (RWS *pre.dlr*: 0.96 ± 0.09 m/s; RWS *post.dlr*: 0.84 ± 0.08 m/s; changes in RWS: 0.12 m/s; t_915.9_ = -7.33, *p* < 0.001), *post.home(R* + *28)* period (RWS *pre.dlr*: 0.96 ± 0.09 m/s; RWS *post.home(R* + *28)*: 0.99 ± 0.21 m/s; changes in RWS: 0.03 m/s; t_916.9_ = 2.02, *p* = 0.044), *post.home(R* + *90)* period (RWS *pre.dlr*: 0.96 ± 0.09 m/s; RWS *post.home(R* + *90)*: 1.00 ± 0.19 m/s; changes in RWS: 0.04 m/s; t_917.6_ = 2.6, *p* = 0.01), in the group cAG (t_21.2_ = 2.31, *P* = 0.031), in *post.dlr.days* (t_916.1_ = 4.02, *p* < 0.001) and in the intercept offset in the *post.dlr* phase for the group cAG (t_915.5_ = -2.53, *p* = 0.012). Please see Fig. 1 and Table 1 for an overview of the RWS values per group and period, and Table 2 for summary statistics of the lme model.

**Table 2:** Summary of the lme model fitted to the real-world walking speed data.

Variables	Estimate	DoF	*t*-value	*p*-value
Intercept	0.932	23.97	38.77	**<** **0.001**
period_*pre.home*	0.000	917.61	−0.01	0.994
period_*post.dlr*	−0.175	915.89	−7.33	**<** **0.001**
period_*post.home(R* + *28)*	0.029	916.86	2.02	**0.044**
period_*post.home(R* + *90)*	0.037	917.62	2.6	**0.01**
group_*iAG*	0.016	21.21	0.48	0.638
group_*cAG*	0.074	21.23	2.31	**0.031**
post.dlr.days	0.009	916.15	4.02	**<** **0.001**
offset.int.cAG.post.dlr	−0.060	915.53	−2.53	**0.012**
offset.int.iAG.post.dlr	0.029	916.13	1.18	0.236

*T*-tests use Satterthwaite’s method. Bold font is used to highlight variables with *p*-value < 0.05. *DoF* Degrees of freedom. Base levels of the model terms are *pre.dlr* for the period variable and *Ctrl* group for the variable group (Group name *Ctrl* refers to the control group, which did not receive any type of intervention to counteract the effects of bed-rest).

The group name *iAG* refers to the group that received intermitted artificial gravity training sessions as intervention to counteract the effects of bed-rest. The group name *cAG* refers to the group that received continuous artificial gravity training sessions as intervention to counteract the effects of bed-rest. The phase names refer to the different phases of the study: *pre.home* refers to a 1-week measurement from the 28th to the 21st day at home prior the bed-rest study; *pre.dlr* refers to a 2-week measurement in the DLR ward in the 14 days prior the bed-rest study; *post.dlr* refers to a 2-week measurement in the 13 days after the bed-rest study; *post.home(R* + *28)* refers to a 1-week measurement from the 21st to the 28th day after the bed-rest; *post.home(R* + *90)* refers to a 1-week measurement from the 83rd to the 90th day after the bed-rest.

Model summary of lme model fitted to RWS data.

### Exploratory analysis

**Fig. 2:**
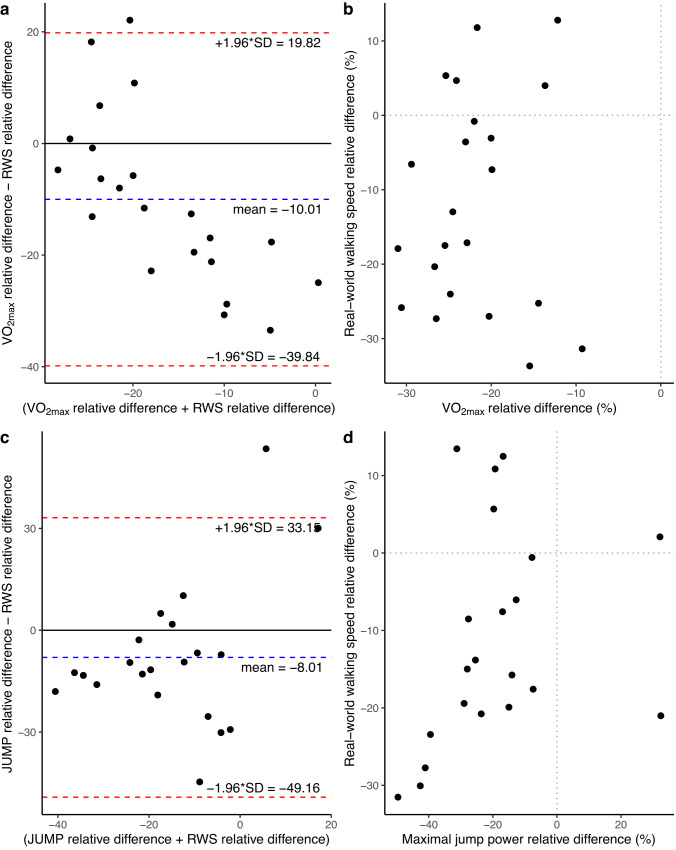
Bland-Altman and correlation plot of the relative differences between post/pre bed-rest. **a** Bland-Altman plot for level of agreement between relative pre/post bed-rest changes of VO_2max_ and RWS. On the *x*-axis are shown the mean of the values and on the *y*-axis are shown the differences between the relative values. **b** Scatterplot showing the correlation between relative pre/post bed-rest changes of VO_2max_ and RWS. On the *x*-axis are shown the VO_2max_ relative changes and on the *y*-axis are shown the RWS relative changes. **c** Bland-Altman plot for level of agreement between relative pre/post bed-rest changes of maximal jump power vs. RWS. On the *x*-axis are shown the mean values and on the *y*-axis are shown the differences between the relative values. **d** Scatterplot showing the correlation between relative pre/post bed-rest changes of maximal jump power and RWS. On the *x*-axis are shown the maximal jump power relative changes and on the *y*-axis are shown the RWS relative changes.

For each of the three parameters (VO_2max_, maximal vertical jumping power and RWS) the relative change was computed and the level of agreement and correlation between (i) VO_2max_ vs. RWS and (ii) maximal vertical jumping power vs. RWS was calculated. The comparison between VO_2max_ vs. RWS showed no agreement (mean bias = -10.01%, upper and lower limit of agreement = 19.82%, -39.84%) and no correlation (t_20_ = 0.24, *p* = 0.81, r: 0.05). Similarly, the comparison of maximal vertical jumping power against RWS showed little agreement (mean bias = -8.01%, upper and lower limit of agreement = 33.15%, -49.16%) and no correlation (t_19_ = 1.41, p = 0.173, r: 0.31) (please see Table 3 for Bland-Altman plot and correlation statistics). Moreover, the Bland-Altman plot in Fig. 2a showed a negative correlation (r: -0.71) between the mean of the two measures (VO_2max_ and RWS) and the difference of the two, suggesting an unequal variance between the measurements (variance VO_2max_ = 35.0, variance RWS = 205.8). After checking normality of distribution of the two measures, a *F*-Test for equality of variances^25^ was performed showing a significant difference (F_21_ = 0.17, *p* < 0.001).

**Table 3:** Bland-Altman plot and correlation analysis statistics for the comparisons of the relative changes post bed-rest in RWS vs. VO_2max_ and RWS vs. JUMP.

Parameter	VO_2max_	JUMP
Mean Bias	−10.01%	−8.01%
Upper limit of agreement	19.82%	33.15%
Lower limit of agreement	−39.84%	−49.16%
Pearson’s r correlation coefficient (95% CI)	0.05 (−0.38:0.46)	0.31 (−0.14:0.65)
Degree of freedom	20	19
*t*-value	0.24	1.42
*p*-value	0.81	0.17

Upper and Lower limits of agreement are calculated as mean bias ± 1.96 St. Dev. of the differences between measurements.

Bland-Altmann and correlation analysis statistics.

Lastly, all the three groups showed a significant decrease of VO_2max_ after bed-rest with no significant differences between groups observed in the loss of VO_2max_ (mean ± sd of differences in percentage pre/post bed-rest per group: Ctrl: -23.8 ± 7.3; iAG; -20.3 ± 4.8, cAG; -21.8 ± 5.7). Similarly, also in maximal vertical jumping power all the three groups had a significant decrease after the bed-rest, although the decrease was more accentuated in the Ctrl group (mean ± sd of differences in percentage pre/post bed-rest per group: Ctrl: -24.5 ± 27.1; iAG; -20.2 ± 10.5, cAG; -12.7 ± 22) (please see Table 4 for absolute and relative values of VO_2max_ and JUMP before and after bed-rest). Results about VO_2max_ and maximal vertical jumping power are originally described elsewhere^26^ to a greater extent than what was reported in this section.

**Table 4:** Mean ± standard deviation of the absolute and relative values before and after bed-rest for the parameters VO_2max_ and maximal jump power (JUMP).

VO_2max_			
	Absolute (L/min)	Relative (%)	*p*-value
**iAG**			
pre bed-rest	3.2 ± 0.6	−	**<** **0.001**
post bed-rest	2.4 ± 0.3	−23.8 ± 7.3	
**cAG**			
pre bed-rest	2.6 ± 0.7	−	**<** **0.001**
post bed-rest	2 ± 0.5	−20.3 ± 4.8	
**Ctrl**			
pre bed-rest	2.8 ± 0.8	−	**<** **0.001**
post bed-rest	2.1 ± 0.6	−21.8 ± 5.7	
**JUMP**			
	Absolute (kW)	Relative (%)	*p*-value
**iAG**			
pre bed-rest	3.9 ± 0.7	−	**0.02**
post bed-rest	2.9 ± 1.2	−24.5 ± 27.1	
**cAG**			
pre bed-rest	2.9 ± 0.8	−	**<** **0.001**
post bed-rest	2.4 ± 0.8	−20.2 ± 10.5	
**Ctrl**			
pre bed-rest	3.4 ± 1.1	−	**0.02**
post bed-rest	3.1 ± 1.6	−12.7 ± 22	

Significance levels are also calculated for each before/after bed-rest comparison and reported. Bold font is used to highlight variables with *p*-value < 0.05 and to highlight the groups name.

Absolute and relative values of VO_2max_ and maximal jump power (JUMP).

## Discussion

The objective of this bed-rest study was to simulate conditions similar to microgravity in space in order to investigate the effects of artificial gravity exposure as countermeasure to bed-rest. By separating the subjects into three groups, which were exposed to intermittent, continuous, or no artificial gravity at all, it was intended to gain insight into possible benefit of artificial gravity as a countermeasure to human physical decondition in space. Results have shown that a decrease occurred in all three groups in each of the parameters studied in this work (RWS, VO_2max_ and vertical jump power), with very little difference between the two intervention groups and the control group. Even though the number of participants was relatively low and gender distribution among subjects was not completely even, the data obtained provided valuable information to address our research questions.

The continuous measurement of RWS opens up possibilities in addition to conventionally used, discrete measurements such as vertical jump power or VO_2max_ measurements^27,28^. First, continuous measurements of RWS, in both controlled and “at-home” environment, offers continuous insight into a patient’s functionality and its recovery process over a longer period of time. In this study, it was shown a high level of compliance, measured as wearing time, not only during the two phases in the DLR ward but also in the “at-home” phases. Although it is difficult to state an absolute threshold, wearing times > 10 h/day are often considered as representative of habitual exposure^29^, and wearing times in the order of 12 or 14 h/day are close to the time that people are awake. This, in combination with laboratory gait tests, would allow to not only gain insight into the *can-do* walking speed, but also into the *do-do* walking speed, which adds a measure of real-word exposure, and thus of greater ecological validity to the laboratory-assessed walking speed^30^.

**Fig. 3:**
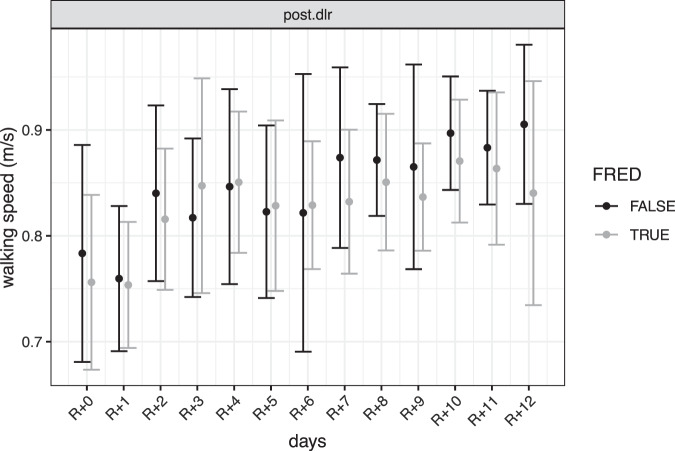
FRED intervention effect on average Real-world Walking Speed (RWS). Average Real-world Walking Speed (RWS) values per FRED training group and day during the *post.dlr* period. Speed values are expressed in m/s. Error bars represent ±1 standard deviation. Group name *TRUE* refers to the group of participants who underwent the FRED training. The group name *FALSE* refers to the group of participants who did not undergo the FRED training. The plot facet name refers to the phase name in which FRED training occurred, the *post.dlr* period which refers to a 2-week measurement in the 13 days after the bed-rest study.

The main findings from the lme model are that RWS decreased after bed-rest, with an average decrease of 13.2% for Ctrl, 10.6% for iAG and 15.2% for cAG compared to the average RWS values observed in the *pre.dlr* phase. Interestingly, the lme showed a significant difference in RWS between *pre.dlr* phase and the two *post.home* phases, suggesting a full recovery and a subsequent improvement in RWS in the periods at home. However, it has to be noted that in the *pre.dlr* phase subjects were confined in the DLR ward, where they had a fixed daily schedule and more days of recordings compared to the two *post.home* phases (13 days of recording in the *pre.dlr* phase vs. 7 days of recording in the *post.home* phases). Moreover, as this improvement is seen in all the three groups, it cannot be attributed to the different training interventions. Interestingly, the cAG group showed on average greater RWS compared to the Ctrl group regardless of the study phase, as faster RWS is observed in all the study phases except for the *post.dlr* phase. These differences are likely explained by the random assignment of participants in the groups, rather than training interventions as faster RWS is also observed in the *pre.home* phase, where no treatment was administered to the intervention groups. During the recovery period (*post.dlr* phase), all the three groups showed a consistent recovery of RWS over time, with an average RWS of 0.82 ± 0.09 m/s for Ctrl, 0.86 ± 0.08 m/s for iAG and 0.83 ± 0.09 m/s for cAG group. However, the group cAG showed a slower recovery of RWS in the *post.dlr* phase compared to the Ctrl group, with an average daily rate of change of RWS of 0.007 m/s for the cAG group compared to 0.008 m/s for the Ctrl group. This is an interesting observation as in all the other study phases, the cAG group showed a greater RWS compared to the Ctrl group; however, the observed slower recovery of RWS of the cAG group in the *post.dlr* phase can be seen as further confirmation that the training interventions used in this study did not help to prevent the reduction nor subsequent recovery of RWS in the recovery phase. Lastly, subjects who underwent FRED training during the *post.dlr* phase did not show any significance difference compared to subjects that did not underwent FRED training, suggesting little impact of the training on the recovery course of RWS (see Fig. 3 to daily RWS values by FRED training group).

Changes in RWS observed after the bed-rest should be considered with caution. On one hand, they could be considered clinically meaningful according to works done by Perera and Kwon^31,32^, where the authors demonstrated that changes in RWS of 0.05 m/s are to be considered clinically meaningful in older adults. In our study, the participants showed on average a RWS decrease from *pre.dlr* to *post.dlr* of 0.13 m/s, a 13% decrease from the initial value (Ctrl: 0.12 m/s, 13.2% decrease; iAG: 0.10 m/s, 10.6% decrease; cAG: 0.15 m/s, 15.2% decrease), values well above the threshold suggested by Perera in his work from 2006^31^ to start considering those changes clinically meaningful. However, on the other hand, it is not totally clear in this type of setting (immobilization by bed-rest and young participants) what these changes represent. Do they reflect a real deconditioning or are they observed mainly because the participants have been more cautious in walking after bed-rest to e.g., reduce the risk of stumbling? It is safe to think that it can be attributed to a combination of all those elements, although reduction in gait speed was also observed in shorter bed-rest studies^33^ to a very similar extent than what observed in this study. This observation is of interest as it is well known that muscle deconditioning in shorter bed-rest studies is less severe than in longer bed-rest studies^34–36^, suggesting that decreases in walking speed in these settings can be partially attributed to factors that goes beyond the mere muscle deconditioning. However, as bed-rest studies that included measurement of walking speed (either under laboratory conditions and/or in real-world settings) are scarce, to further answer these questions, additional bed-rest studies would be needed where RWS is measured together with laboratory gait tests to also understand the correlation and agreement between those two variables and see whether the functional ability to walk at either chosen speed or maximal speed is impaired at the same magnitude at which the RWS is impaired.

Kuspinar^37^ previously investigated the predictability of VO_2max_ data with submaximal tests including the vertical jump test as well as mean walking speed in patients suffering from multiple sclerosis. In that study, the 6-min walking test (6MWT) as a submaximal effort test had the highest, though still weak, correlation with absolute values of VO_2max_. The exploratory analysis in the present study pertaining the level of agreement between changes in VO_2max_ and RWS before and after bed-rest, and maximum vertical jump power and RWS before and after bed-rest, showed that changes in VO_2max_ and JUMP values also have very little to no agreement and correlation with RWS. Although both walking and cycling (and thus the VO_2max_ test on the cycle ergometer) involve regular distinct phases that alternate bilaterally, the VO_2max_ test on the cycle ergometer involved bursts of incremental effort until exhaustion with much greater muscle activation that cannot be sustained for a prolonged time, and therefore it is used to assess only the cardiorespiratory fitness. On the other hand, walking at self-selected speed is a low-effort movement that can be sustained for a prolonged period, which results in a combination of multiple factors, such as metabolic and coordination factors and muscles power, thus it should be expected that the two will assess different types of fitness. This is supported by Abellan van Kan^12^ who also postulated that individual’s self-selected or usual walking speed is indicative of current functional status and numerous health outcomes in older adults. However, in this study, it was preferred to assess the VO_2max_ with a cycle ergometer rather than a treadmill, although it is known that VO_2max_ assess via treadmill is generally higher than the VO_2max_ assessed via a cycle ergometer^38–40^. Assessing the VO_2max_ via cycle ergometer was chosen mainly due to safety reasons as it is safer to use following bed-rest and it allows better standardization and data robustness due to fewer artifacts coming from the upper body motion. Similarly, this applies also to the comparison of walking speed and JUMP. The latter is a test of maximal neuromuscular power with most of the movement exerted in the vertical direction, while during walking most of the movement acts predominantly on the horizontal direction, so it is not surprising that the two assess different types of fitness.

Importantly, the findings of these studies indicate that measurements from one submaximal test such as the daily average walking speed are insufficient for reliably predicting a maximal test such as the VO_2max_ measurements, but also vice-versa. Furthermore, walking speed, not walking distance, was shown to be a robust outcome variable, as shown by a study involving patients suffering from multiple sclerosis^18^. In light of the above, continuous monitoring of RWS can provide additional insight into a person’s recovery process, in combination with others, more established functional tests (e.g., VO_2max_) to monitor the recovery state of individuals after bed-rest or after a prolonged space flight, where the multifactorial syndrome with disturbance in gait patterns is experienced by many astronauts upon return to Earth^41^. Lastly, if it can be fully demonstrated that the loss and the subsequent recovery of RWS does reflect in part the health status of individuals in populations with characteristics similar to those observed in astronauts, it opens up possibilities in the monitoring of the recovery process of astronauts upon return from mid to long space flights, as daily RWS values are easy to obtain, very unintrusive as it was shown by the high level of acceptance and they offer a robust outcome hardly influenced by potential outliers (as opposed to other functional tests performed on single test days—e.g., VO_2max_ of maximal vertical jump test, where the influence of one outlier has a greater impact on the final outcome and its interpretation).

As it is typically the case for bed-rest studies, in the present study, sample size was rather small, limited to 24 participants with 8 participants per group. There were also limitations that were specific to the present bed-rest study. Following the bed-rest, subjects were instructed to use a wheelchair on R + 0 and R + 1. Nonetheless, many physically challenging tests were performed on the first day of re-ambulation and subjects would likely have had muscle soreness from the acute reloading. It also seems reasonable to assume that RWS during the early recovery phase could have been influenced by the leg muscle biopsy that was obtained at the end of bed-rest. Additionally, during the *post.dlr* period, subjects walked to and from experiments, together with the facility personnel. There is a possibility that the subjects’ chosen RWS was conditioned by the accompanying person, although this seems unlikely as the DLR staff adjusted their walking speed to match the subject’s walking speed.

Another limitation of this study is the *stepwave* algorithm used to estimate RWS. Keppler^42^ originally proposed and validated the *stepwave* algorithm on an average older population than what was presented in this study. Even though walking parameters are naturally affected by age, the *stepwave* algorithm did proof to be relatively robust against aging as shown by Wiedmann in her study^43^, where it was shown that the algorithm was able to reliably detect the presence of gait in a pediatric population with or without cerebral palsy. Moreover, actibelt® was chosen as it has a track record of scientific literature supporting that the platform used to gather accelerometry data and convert them to actimetry data is reliable and used in clinical settings and space research (refs. ^44–46^^,^^30^^,^^47–49^).

Finally, it would have been interesting to compare RWS with a standard lab-based walking test, which, however, was not implemented as part of this bed-rest study.

It was shown that RWS decreases with bed-rest to an extent that in different populations (e.g., elderly population or frail populations) is clinically meaningful. Even though the decreases in RWS observed here do not necessarily have immediate health implication, it is a remarkable finding in itself that RWS is sensitive enough to reflect a deconditioned physique in healthy young/middle-aged individuals. For future studies, it would be of interest to investigate how the deconditioning and recovery process of RWS compare against laboratory gait speed tests. It is concluded that, given the fact that RWS can be obtained unobtrusively, at a low operational cost and at a low risk, it can be considered as a functional relevant tool to continuously monitor the recovery process of individuals after bed-rest and other deconditioning processes.

## Methods

### Study design

**Fig. 4:**
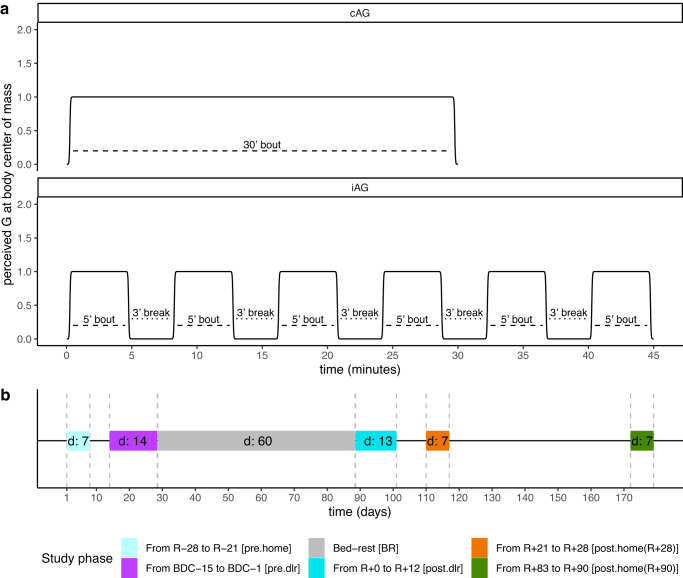
Visual representations of the training sessions and the study phases. **a** Schematic of the training sessions used as countermeasure to bed-rest for the two training groups. On the *x*-axis is represented the elapsed time in minutes, on the *y*-axis is shown the acceleration of gravity (G) perceived at the body center of mass. In the top plot is shown the schematic representing the training sessions of the continuous artificial gravity (cAG) group. On the bottom plot is shown the schematic representing the training session of the intermittent artificial gravity (iAG) group. Both groups had daily training sessions. The cAG was exposed to artificial gravity for 30 consecutive minutes, while the iAG group was exposed to artificial gravity for six 5-min bouts, with a 3-min break in between the bouts. **b** Schematic of the study phases in which continuous tri-axial accelerometer measurements were acquired. *X*-axis represents time in days, and vertical dashed lines represent start and end of each of the study phases. Text in colored bars denotes the duration of the phase. Study phase abbreviations are shown in squared brackets after the name period in the top of the diagram.

This bed-rest study was performed at the *:envihab* facility of the German Aerospace Center (Deutsches Zentrum fur Luft- und Raumfahrt, DLR). The study was conducted in two separate campaigns with twelve participants in each campaign. For each subject, each campaign lasted 89 days, which consisted of a 15-day baseline data collection (*BDC*), 60-day 6° head down tilt (*HDT*), and a 14-day recovery (*R* + ) phase. During the ambulatory stationary phases of the study, the subjects stayed in the controlled environment of the DLR facility. Artificial gravity (AG) exposure was presented as a potential countermeasure and was provided by means of horizontal centrifugation during the *HDT* phase with a 3.8 meters radius short-arm human centrifuge. Daily, the participants were exposed to 30 min of centrifugation at 1 G at the center of mass and ~2 G at the feet. The 30-min AG intervention was completed either in one continuous 30-min run (cAG), or intermittently with 3-min breaks in between six 5-min bouts of centrifugation (iAG) (see Fig. 4a for illustration of training sessions). Participants were positioned on one arm of the human-centrifuge, with their feet pointing outwards. All sessions were supervised by a medical doctor. During the ambulatory phases (*BDC* and *R*+), physical activity was restricted to free movement within the ward and to standardized reconditioning sessions. Additionally, half of the participants underwent 30 min of Functional Re-adaptive Exercise Device (FRED) training every day during the *R* + (recovery) phase. Prior to the study, subjects were assigned to the FRED group in a stratified manner to ensure that experimental group allocation and FRED Training were balanced. Subjects who underwent FRED Training were selected from both campaigns (six subjects from campaign 1, and six subjects from campaign 2).

### Subjects

In total, 24 healthy participants were recruited from the general public to participate in the study. The recruitment of the subjects included methods, such as use of test subject archives, announcements in electronic media and the internet. A participant was deemed to be healthy after undergoing thorough physical and psychological examinations. Physical examinations included resting and stress electrocardiogram, orthostatic testing, lung function and eye examinations as well as a medical anamnesis, followed by blood test for HIV, hepatitis, tuberculosis, and thrombophilia. The psychological screening consisted of an initial preliminary psychological evaluation, the Freiburger Persönlichkeitsinventar (FPI) personality checklist. Candidates then underwent detailed psychological screening that involved additional questionnaires; the Temperament Structure Scale and the 60-item NEO-FFI personality inventory, along with the subject’s self-reported biography and a concluding in-person interview.

On the first day of the *HDT* intervention, the recruited subjects were randomly assigned to either the continuous centrifugation group (cAG, *n* = 8, age 32 ± 10 years, height 173 ± 8 cm, body mass 72 ± 10 kg, 3 female), intermittent centrifugation group (iAG, *n* = 8, age 34 ± 11 years, height 174 ± 11 cm, body mass 71 ± 5 kg, 3 female) or the control group (CTRL, *n* = 8, age 34 ± 8 years, height 177 ± 7 cm, body mass 79 ± 13 kg, 2 female), please see Table 5 for summary statistics of participants’ anthropometric and demographic data, and significance levels of differences between groups. Prior to commencing the study, all participants gave written informed consent to the experimental procedures, which were authorized by the ethics committee of the Northern Rhine Medical Association (Ärztekammer Nordrhein) in Duesseldorf, Germany. The subjects received financial compensation for their participation. The study is registered at the German Clinical Trials Register (www.drks.de) under the number DRKS00015677.

**Table 5:** Summary statistics of participants’ anthropometric and demographic data.

Characteristics	N	Ctrl, *N* = 8	cAG, *N* = 8	iAG, *N* = 8	*p*-value^a^	*q*-value^b^
Gender, n / N (%)	24				> 0.9	> 0.9
Females		2 / 8 (25%)	3 / 8 (38%)	3 / 8 (38%)		
Males		6 / 8 (75%)	5 / 8 (62%)	5 / 8 (62%)		
Age, Mean ± SD	24	34.2 ± 7.9	31.9 ± 9.7	33.8 ± 10.8	0.7	0.9
Height (cm), Mean ± SD	24	177.0 ± 7.3	172.5 ± 8.0	174.1 ± 10.5	0.4	0.8
Weight (kg), Mean ± SD	24	79.4 ± 12.7	71.8 ± 10.2	71.4 ± 4.5	0.087	0.3

^a^ Fisher’s exact test for categorical variables and Kruskal–Wallis rank sum test for continuous variables.

^b^ Benjamini & Hochberg correction for multiple testing.

Summary statistics of participants.

### 3D-accelerometry

During the entire *BDC* and *R+* periods in the facility, subjects were equipped with a tri-axial accelerometer (actibelt®, Trium Analysis Online GmbH, Munich, Germany) positioned on the frontal region, below the umbilicus. The device is rechargeable, portable in size and records the accelerations in the three dimensions with a sample frequency of 100 Hz. The participants kept the actibelt® on for the duration of the *BDC* phases (from *BDC-28* to *BDC-21*, and from *BDC-15* to *BDC-1*), and again during three distinct recovery phases (from *R* + *0* to *R* + *12*, from *R* + *21* to *R* + *28* and from *R* + *83* to *R* + *90*). The participants were instructed to remove the belts only for all MRI experiments, and when taking a shower or when sleeping. Thus, actibelt® was used during five distinct periods throughout the study (see Fig. 4b for overview and phases abbreviation names and schematic of study phases in which actibelt® measurements were acquired). For each period, actibelt® wearing time was assessed via an electronic switch of the device that senses physical closure of the buckle.

### VO_2max_ measurements

Maximum oxygen uptake capacity (VO_2max_) was assessed by means of a maximal cycle ergometry (Lode Excalibur, Groningen, The Netherlands) once before (BDC-3) and once immediately following bed-rest (R + 0) in an environmentally (temperature/humidity) controlled laboratory. Ambient temperature and humidity were controlled by AC units, while ambient pressure was used to calibrate the cycle ergometry together with the ambient temperature at the time of the measurements. The measurement protocol consisted of 5 min of seated rest on the cycle ergometer followed by 3 min of pedaling at a cadence of 75 rpm at 50 Watts. Subsequently, the power was increased by 25 Watts every minute, starting from 3 min at 50 W, until voluntary exhaustion under strong verbal encouragement. Following voluntary exhaustion, subjects continued to pedal for 5 min at a low work rate to allow for an active recovery. Subjects were asked to rate their perceived exertion on a 6-20 point Borg scale and continuous (breath-by-breath) systemic oxygen uptake (VO_2_) and carbon dioxide (CO_2_) emission were obtained using the Innocor system (Innovision, Odense, Dänemark). Moreover, a 12-lead ECG (Padsy, Medset Medizintechnik, Germany) was used to continuously monitor and record the heart rate. Lastly, spiroergometric data were filtered by calculating the median of 5 breaths and the moving average over 30 s and the peak values for the VO_2max_ values were calculated. For statistical analyses, Delta values were calculated, and given in percent of the BDC-value.

### Vertical jump measurements

Before (BDC-8) and after (R + 0) the bed-rest period, subjects performed maximal countermovement jumps. Peak vertical jump power (Pmax) was calculated from the time course of ground reaction forces (Novotec Medical GmbH, Pforzheim, Germany). Subjects were familiarized with the test in a separate session before the baseline measurements. Before the maximal vertical jump attempt, subjects warmed-up by means of three warm-up squats and then were instructed to practice three countermovement jumps at roughly half of maximum effort to ensure that the test instruction was correctly understood. Thereafter, subjects performed three maximal countermovement jumps with short breaks in between. Pmax (in kW) was chosen as the highest value of the three trials. For statistical analyses, Delta values were calculated, and given in percent of the BDC-value.

### Data processing

Accelerometry data were uploaded to the actibelt® data warehouse and checked for completeness. For each subject, a total of 48 days with at least 10 h/day of recording time each were expected (making up for a total of 1152 expected measurements). Data were subsequently analyzed, and gait speed, daily number of steps and wearing time calculated with the *stepwave* algorithm^42^ along with other gait parameters were retrieved. The *stepwave* algorithm calculates mean speed per walking step, thus, RWS is calculated as the average of the mean speed per walking step in a given day. To address the main hypothesis, a decrease in RWS after the bed-rest period with a subsequent recovery to the initial RWS values measured before the bed-rest, walking speed was selected as the principal parameter for the analysis. As a first step we manually investigated the completeness of the retrieved data with respect to the phases and neglected data which were recorded outside the scheduled phases. Adherence is a parameter which represents the number of hours the actibelt® was worn by the individual subjects. Data with adherence below 1 h/day were omitted. Measurements with < 10 h/day of recording time for a given day were marked as incomplete. For the *pre.home* period a visual inspection of the walking speed data was performed in order to ensure that the notable difference between the group cAG and the other two groups, iAG and Ctrl, did not originate from recording issues.

VO_2max_ data and maximal vertical jump power data were loaded into R Studio and distinctly merged with the walking speed data using as a merging key the subject id and the date. This implies that the daily RWS used as baseline to calculate the relative difference is the daily RWS calculated at the day of the VO_2max_ test for the comparison RWS vs. VO_2max_, and the daily RWS calculated at the day of the maximal vertical jump power test. Out of the three maximal countermovement jumps, we selected the jump that showed the highest power output. Once the three datasets were merged, the relative changes after bed-rest were calculated with the formula $100* \frac{{B}_{x}-{A}_{x}}{{A}_{x}}$, where A denotes the baseline value of *x*, B denotes the values of *x* after bed-rest and *x* denotes the type of data used, either RWS data, VO_2max_ data or maximal vertical jump power data. Thus, negative values will denote a decrease in performance after bed-rest from baseline values, and vice versa, positive values will denote an improvement in performance after bed-rest from the baseline values.

### Statistical analysis

Statistical analysis was performed using R Studio v. 4.0.3 (RStudio: Integrated Development Environment for R, RStudio, Inc., Boston, MA). It included: an assessment of whether the incomplete measurements were missing data at random, fitting of a linear mixed effect (lme) model and pruning of variables with low statistical significance.

A common approach to dealing with incomplete observations is to remove them from the analysis. However, this process may introduce bias, and it wastes valuable partial data. Instead, it was assessed if the incomplete measurements are missing data at random by randomly removing segments of measurement from the complete observations (measurements with ten or more hours of recording per day) and compare the two distributions to determine whether the data are missing at random or not (please see Supplementary Methods and Supplementary Figures for more details on the procedure). Compelling evidence was not found to conclude that the data were not missing at random, and thus, they were included in further analysis.

Denote the indication function with:

1 $${\chi }_{p}(t):=\left\{\begin{array}{l}1,when\,t\in p,\\ 0,else,\end{array}\right.$$

Where either: *t* is time in days and *p* is a period or *t* is a patient index and *p* is a patient group. The lme model for subject *i* and day *t* took the following form:

2 $$\begin{array}{c}RW{S}_{i}(t)={\beta }_{0}+({\beta }_{1,1}{\chi }_{pre.home}(t)+{\beta }_{1,2}{\chi }_{post.dlr}(t)+{\beta }_{1,3}{\chi }_{post.home(R+28)}(t)\,\\ \,+{\beta }_{1,4}{\chi }_{post.home(R+90)}(t))+({\beta }_{2,1}{\chi }_{cAG}(i)+{\beta }_{2,2}{\chi }_{iAG}(i))+{\beta }_{3}post.dlr.days(t)\\ \,+{\beta }_{4}offset.int.cAG.post.dl{r}_{i}(t)+{\beta }_{5}offset.int.iAG.post.dl{r}_{i}(t)+{{\epsilon }}_{i}(t)\end{array}$$

Where:

${post}.{dlr}.{days}(t):=[t-{t}^{* }+1]{\chi }_{{post}.{dlr}}(t),$ *a*nd t* is the first day of the *post.dlr* period,

$${{offset}.{int}.{cAG}.{post}.{dlr}}_{i}(t):={\chi }_{{post}.{dlr}}(t){\chi }_{{cAG}}(i),$$

$${{offset}.{int}.{iAG}.{post}.{dlr}}_{i}(t):={\chi }_{{post}.{dlr}}(t){\chi }_{{iAG}}(i),$$

${\epsilon }_{i}(t) \sim {\rm{{\rm N}}}(0,{\rm{\sigma }})$ and all *β* coefficients above are reals.

The term *post.dlr.days* is a vector containing numbers representing the elapsed days in the *post.dlr* period since *R* + *0*. The terms *offset.int.cAG.post.dlr* and *offset.int.iAG.post.dlr* are the terms to offset the group intercepts in the *post.dlr* phase. Those two terms have been introduced to account for group differences in RWS that might occur randomly, and not related to the study interventions, and thus interfere with the interpretation of the group RWS recovery. Thus, the intercept in the *post.dlr* phase was set to 0.

The model formula was implemented using the command *lmer* from the R package *lmerTest*, using Satterthwaite’s method for computing the *t*-tests^50^.

This model formula was chosen using a simplification strategy, removing model terms whenever showing not significant importance in the model. Significance of model terms was determined using Satterthwaite approximation^50^. The initial model additionally contained one linear term to account for those subjects that underwent FRED training every day during the recovery phase within the DLR ward (from R + 0 to R + 12); an interaction term between *group* and *post.dlr.days*; and additional interaction term between *group* and *period*. However, the linear term and both interaction terms did not show any significance in the model formula and were thus pruned away.

Lastly, Bland-Altman plots^51^ and correlation analysis using Pearson’s product moment correlation coefficient was done aimed at finding out the level of agreement and correlation between changes in daily RWS between pre/post-bed-rest and (i) changes in VO_2max_ pre/post-bed-rest and (ii) the maximal power exerted during vertical jump between pre/post-bed-rest^52,53^.

### Reporting summary

Further information on research design is available in the Nature Research Reporting Summary linked to this article.

### Supplementary information

Supplementary material

Reporting Summary

## Data Availability

The datasets used for producing the current work are available in the Open Science Framework repositories agbresa2019_rws/data_frames and agbresa2019_rws/input under the following link https://osf.io/3rqex/?view_only=fa4b5b086d8e41eb9cf8a3c473b81ad4.

## Code availability

The code used for producing the current work is available in the Open Science Framework repository agbresa2019_rws/Rscripts under the following link https://osf.io/3rqex/?view_only=fa4b5b086d8e41eb9cf8a3c473b81ad4.
