# Supplementary material for: Changes in real-world walking speed following 60-day bed-rest
Source: NPJ Microgravity. 2024 Jan 13;10:6. doi: 10.1038/s41526-023-00342-8 (PMC10786829; doi:10.1038/s41526-023-00342-8)
Supplement: Supplementary file 1 — Supplementary material [file 41526_2023_342_MOESM1_ESM.pdf]

Changes in Real-world walking speed following 60-day bed-rest

Supplementary Methods

Denote by $\mathbb{P}^{(i,p,d)}$ the (idealized) probability distribution of the average walking speed of an individual $i \in \{1, \ldots, 24\}$ on a day $d \in \{-28, \ldots, 90\}$ during a period $p \in \{pre.home, \ldots, post.home(R + 90)\}$. (Note that for a given day d, it is possible to uniquely determine the period p during which d happens). Similarly, denote by $\mathbb{P}^{(i,p)}$ the probability distribution of the daily average walking speed of a subject *i* during a period *p*. We make the following assumption:

Assumption 1: $\mathbb{P}^{(i,p,d)} \equiv \mathbb{P}^{(i,p)}$, i.e., the distribution of the daily average walking speed of an individual *i* conditionally on the period *p* is the same for all days during that period.

Denote by $\mathbb{P}_k^{(i,p)}$ the distribution of the average walking speed of an individual *i* during a period *p*, that is computed based on k random minutes of walking during a single day. Finally, define $\mathbb{P}_{n,k}^{(i,p)}$ to be the distribution $\mathbb{P}^{(i,p)}$ conditional on the subject *i* having worn a belt for *n* hours during the day and having walked for *k* minutes during that time. $\mathbb{P}_{n,k}^{(i,p)}$ for $n < 10$ correspond to the law according to which incomplete observations follow. We are now ready to state the following hypothesis.

Hypothesis 1AP: Distributions $\mathbb{P}_{n,k}^{(i,p)}$ are equal to $\mathbb{P}_k^{(i,p)}$ for all $n < 10$ and all observed k.

In other words, Hypothesis 1AP states that for any individual, the walking speed during the segments of the data that are missing is not statistically different from the walking speed during the segments that were registered.

We will define an informal, joint test as follows: denote by $F_k^{(i,p)}$ the cumulative distribution function (cdf) of the average walking speed computed based on *k* minutes of walking of an individual *i* during period *p* and denote by $\hat{x}_{n,k}^{(i,p,d)}$ the average walking speed of an individual *i* on the day d (with period *p* being determined by day d), with *n* and *k* being only the indicators for the wear time and walking time respectively. We have the following result:

Proposition 1AP: Under Hypothesis 1AP:

$(F_k^{(i,p)})^{-1}(\hat{x}_{n,k}^{(i,p,d)}) \sim Unif([0,1])$ for all *i, p, d, n, k.*

Proof: This follows immediately from the probability integral transform.

We can now define the following algorithm:

Under Hypothesis 1AP, by Proposition 1, the elements of set *S* from Algorithm 1 below (Supplementary Table 1) are distributed approximately as uniform random variables (only approximately, because empirical cdfs are used in place of cdfs). We can exploit this, by looking at the Q-Q plot (Supplementary Figure 1, panel c) that compares the distribution of the elements of *S* with a $Unif([0,1])$. Any deviations from a $x = y$ line would be indicative of Hypothesis 1AP being violated.

Supplementary Tables

Supplementary Table 1

Algorithm 1:
Initialization:
Set $S \leftarrow \emptyset$;
**for** each incomplete observation $\hat{x}_{n,k}^{(i,p,d)}$ **do**
Identify all complete observations $\{\hat{x}^{(i,p,d)}\}_d$ for (i, p);
Compute an empirical cdf $\hat{F}_k^{(i,p)}$ by sub-sampling from $\{\hat{x}^{(i,p,d)}\}_d$;
Evaluate $u^{(i,p,d)} \leftarrow (\hat{F}_k^{(i,p)})^{-1}(\hat{x}_{n,k}^{(i,p,d)})$;
Set $S \leftarrow S \cup \{u^{(i,p,d)}\}$;
**end**
**return** S

Supplementary Figures

Supplementary Figure 1

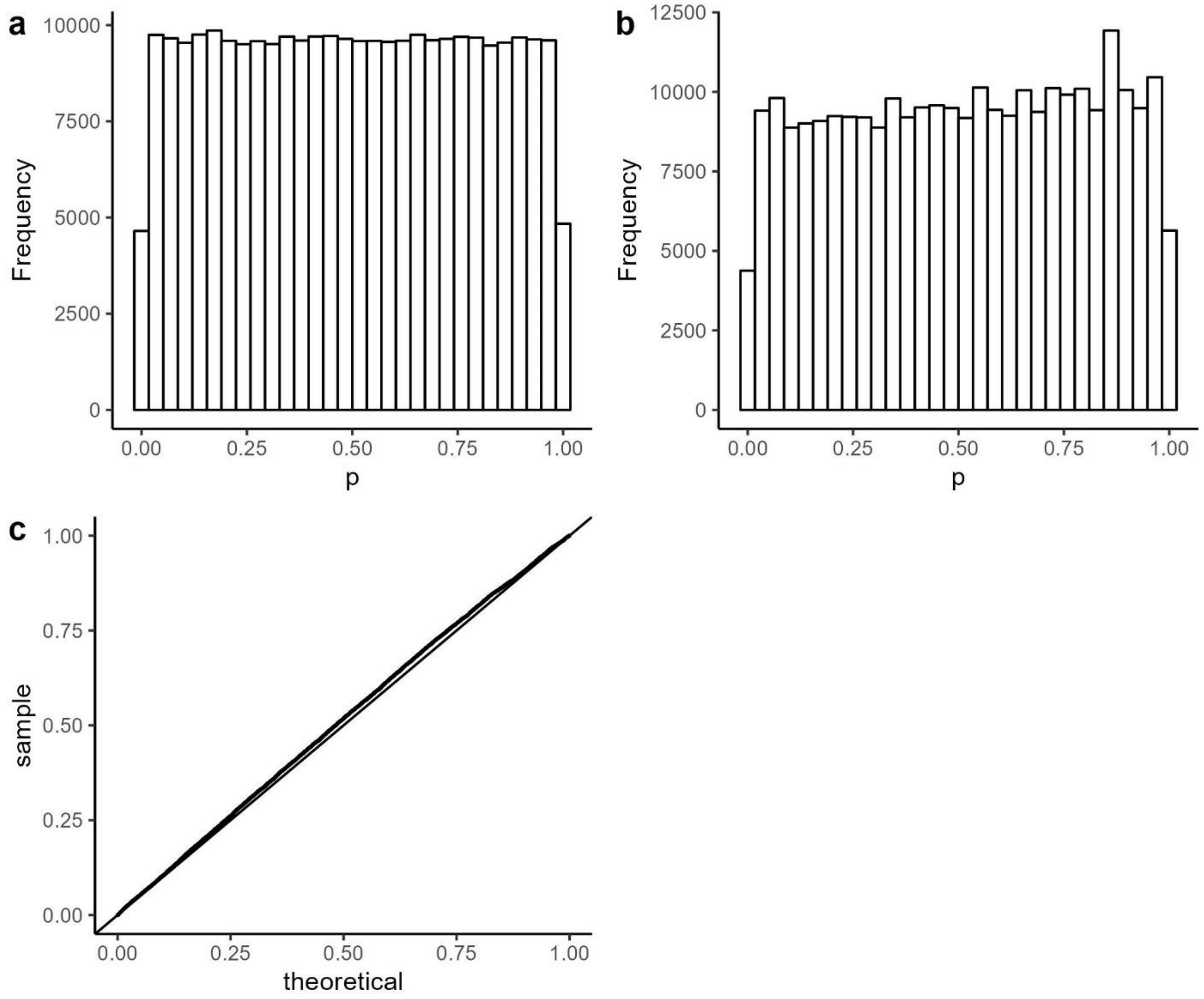

Supplementary Figure 1: Visual checks performed to assess whether the missing data of the incomplete observations were missing at random or not.

Panel a: Histogram of the empirical cumulative distribution function of the theoretical partial observations.

Panel b: Histogram of the empirical cumulative distribution function of the incomplete observations.

Panel c: Q-Q plot of probability distributions of the incomplete observations and theoretical partial observations.
