# Supplementary material for: Changes in real-world walking speed following 60-day bed-rest
Source: NPJ Microgravity. 2024 Jan 13;10:6. doi: 10.1038/s41526-023-00342-8 (PMC10786829; doi:10.1038/s41526-023-00342-8)
Supplement: Supplementary file 2 — Reporting Summary [file 41526_2023_342_MOESM2_ESM.pdf]

nature portfolio

Corresponding author(s): Prof. Dr. Jörn Rittweger
Last updated by author(s): Nov 22, 2023

Reporting Summary

Nature Portfolio wishes to improve the reproducibility of the work that we publish. This form provides structure for consistency and transparency in reporting. For further information on Nature Portfolio policies, see our Editorial Policies and the Editorial Policy Checklist.

Statistics

For all statistical analyses, confirm that the following items are present in the figure legend, table legend, main text, or Methods section.

n/a	Confirmed	
☐	☒	The exact sample size (*n*) for each experimental group/condition, given as a discrete number and unit of measurement
☐	☒	A statement on whether measurements were taken from distinct samples or whether the same sample was measured repeatedly
☐	☒	The statistical test(s) used AND whether they are one- or two-sided<br>*Only common tests should be described solely by name; describe more complex techniques in the Methods section.*
☐	☒	A description of all covariates tested
☐	☒	A description of any assumptions or corrections, such as tests of normality and adjustment for multiple comparisons
☐	☒	A full description of the statistical parameters including central tendency (e.g. means) or other basic estimates (e.g. regression coefficient) AND variation (e.g. standard deviation) or associated estimates of uncertainty (e.g. confidence intervals)
☐	☒	For null hypothesis testing, the test statistic (e.g. *F*, *t*, *r*) with confidence intervals, effect sizes, degrees of freedom and *P* value noted<br>*Give P values as exact values whenever suitable.*
☒	☐	For Bayesian analysis, information on the choice of priors and Markov chain Monte Carlo settings
☒	☐	For hierarchical and complex designs, identification of the appropriate level for tests and full reporting of outcomes
☐	☒	Estimates of effect sizes (e.g. Cohen's *d*, Pearson's *r*), indicating how they were calculated

Our web collection on statistics for biologists contains articles on many of the points above.

Software and code

Policy information about availability of computer code

Data collection	For data collection a tri-axial accelerometer named actibelt, manufactured by Trium Analysis Online GmbH, Minuch, Germany was used
Data analysis	For data analysis R Studio v. 4.0.3 was used

For manuscripts utilizing custom algorithms or software that are central to the research but not yet described in published literature, software must be made available to editors and reviewers. We strongly encourage code deposition in a community repository (e.g. GitHub). See the Nature Portfolio guidelines for submitting code & software for further information.

Data

Policy information about availability of data

All manuscripts must include a data availability statement. This statement should provide the following information, where applicable:

- Accession codes, unique identifiers, or web links for publicly available datasets
- A description of any restrictions on data availability
- For clinical datasets or third party data, please ensure that the statement adheres to our policy

The datasets used for producing the current work are available in the Open Science Framework repository agbresa2019_rws under the following link https://osf.io/3rqex/?view_only=fa4b5b086d8e41eb9cf8a3c473b81ad4.

Research involving human participants, their data, or biological material

Policy information about studies with human participants or human data. See also policy information about sex, gender (identity/presentation), and sexual orientation and race, ethnicity and racism.

Reporting on sex and gender	Sex was considered in the study design and out of 24 participants in the study, 8 were females. Gender was not collected as information to perform the analysis. The finding of this study applies to both sexes.
Reporting on race, ethnicity, or other socially relevant groupings	Socially constructed variables or socially relevant categorization variables have not been used in this manuscript.
Population characteristics	24 participants took part in the study and were randomly assigned to three groups. Average age (± standard deviation) of the participants was 33.3±9.2. Average height was 174.5±8.7 cm and average weight was 75.2±9.5 kg.<br>Out of 24 participants, 8 were females.
Recruitment	Participants were recruited from the general public tand the recruitment included methods such as use of test subject archives, announcements in electronic media and the internet.
Ethics oversight	Ethics committee of the Northern Rhine Medical Association (Ärztekammer Nordrhein) in Duesseldorf, Germany

Note that full information on the approval of the study protocol must also be provided in the manuscript.

Field-specific reporting

Please select the one below that is the best fit for your research. If you are not sure, read the appropriate sections before making your selection.

☒ Life sciences ☐ Behavioural & social sciences ☐ Ecological, evolutionary & environmental sciences

For a reference copy of the document with all sections, see nature.com/documents/nr-reporting-summary-flat.pdf

Life sciences study design

All studies must disclose on these points even when the disclosure is negative.

Sample size	The AGBRESA bed rest study was conducted as an international collaborative effort between the National Aeronautics and Space Administration (NASA), the European Space Agency (ESA), and the German Aerospace Center (DLR). The work presented here was amongst more than 20 research proposals in the fields of cardiovascular, immunology, genetics, muscle and bone, ocular, vestibular, psychology, sleep, cognition, and behavioral performance were integrated into a comprehensive assessment of the long-term responses to AG and bed rest. Of all parameters, reliable detection and quantification of retinal nerve fiber layer thickness (RNFL) during the bed rest period was critical for the success of this study (i.e. RNFL served as the primary endpoint). The sample size of 24 (8 subjects in each of 3 arms) was pre-determined by NASA and ESA. Power analyses showed that this sample size was adequate to detect a difference between groups if the intervention 1 or 2 attenuated the expected increase in RNFL thickness by 50% (or more) compared to the HDT alone, which was hypothesized.
Data exclusions	Two subjects were excluded due to missing baseline data collection
Replication	Replication of findings can be achieved by re-analyzing the raw accelerometric data as described in the manuscript. However, accelerometric signals cannot be exactly reproduced as they recorded study's subjects in their free-living environment without any type of restriction to confine their movements. Thus, it is really unprobeable to being able to recreate the accelerometric signals acquired in this study.
Randomization	On the first day of the Head-Down Tilt phase, subjects were randomly assigned to one of the three groups.
Blinding	*Describe whether the investigators were blinded to group allocation during data collection and/or analysis. If blinding was not possible, describe why OR explain why blinding was not relevant to your study.*

Reporting for specific materials, systems and methods

We require information from authors about some types of materials, experimental systems and methods used in many studies. Here, indicate whether each material, system or method listed is relevant to your study. If you are not sure if a list item applies to your research, read the appropriate section before selecting a response.

Materials & experimental systems

n/a	Involved in the study	
☒	☐	Antibodies
☒	☐	Eukaryotic cell lines
☒	☐	Palaeontology and archaeology
☒	☐	Animals and other organisms
☐	☒	Clinical data
☒	☐	Dual use research of concern
☒	☐	Plants

Methods

n/a	Involved in the study	
☒	☐	ChIP-seq
☒	☐	Flow cytometry
☒	☐	MRI-based neuroimaging

Clinical data

Policy information about clinical studies

All manuscripts should comply with the ICMJE guidelines for publication of clinical research and a completed CONSORT checklist must be included with all submissions.

Clinical trial registration: DRKS00015677 (from www.drks.de)

Study protocol: *Note where the full trial protocol can be accessed OR if not available, explain why.*

Data collection: Subjects were equipped with a tri-axial accelerometer positioned on the frontal region, below the umbilicus. The participants kept the tri-axial accelerometer on for the duration of the baseline data collection phase and again during three distinct recovery phases. Thus, the tri-axial accelerometer was worn by each subject during five distinct periods throughout the study.

Outcomes: Primary and secondary outcomes were defined on the basis of the reasearch questions of interest and the measurements were assessed by mean of statistical analysis.
